# Bilateral hemorrhagic pleural effusion due to kerosene aspiration

**DOI:** 10.4103/0970-2113.80329

**Published:** 2011

**Authors:** Rajendra Prasad, Saurabh Karmakar, Rakhee Sodhi, Shilpi Karmakar

**Affiliations:** *Department of Pulmonary Medicine, Chhatrapati Shahuji Maharaj Medical University (Earlier K.G.M.C.), Lucknow 226 003, U.P, India*; 1 *MBBS, G.S.V.M. Medical College, Kanpur, UP, India*

**Keywords:** Hydrocarbon, kerosene aspiration, pleural effusion

## Abstract

Kerosene ingested, intentionally or accidentally, is toxic. Data is scarce on complications and outcomes of hydrocarbon poisoning following kerosene aspiration in adults and there has been no known case of bilateral hemorrhagic effusion occurring due to it in literature. We, hereby, report a case of a bilateral hemorrhagic pleural effusion secondary to hydrocarbon aspiration in a 40-year old adult.

## INTRODUCTION

Kerosene oil poisoning leading to aspiration is a very uncommon mode of hydrocarbon poisoning in adults. Many hydrocarbons in kerosene like hexane, naphthalene, octane, and phenanthrene are toxic to humans. The most serious side effect is aspiration pneumonia. Hydrocarbon aspiration (HA) can cause significant pulmonary disease by inducing an inflammatory response, hemorrhagic exudative alveolitis, and loss of surfactant function. Secondary effects in the lungs include pneumothorax, pneumatocele, or bronchopleural fistula. We searched for a case of bilateral hemorrhagic pleural effusion occurring in adult following kerosene ingestion but to the best of our knowledge, such a case has not been reported till now. Hence, we are reporting this case.

## CASE REPORT

A previously healthy 40-year old male, presented to our emergency department, with the chief complaints of progressively increasing breathlessness with chest pain of pleuritic nature and high grade fever since 3 days. On initial examination, his respiratory rate was 32/min and oxygen saturation was 88%. His BP was 112/80 mmHg and pulse rate was 98/min. Rest of the general physical examination was within normal limits and there was no cyanosis. Auscultation revealed diminished breath sounds bilaterally. Evaluation of rest of the systems were within normal limits

**Figure 1:**
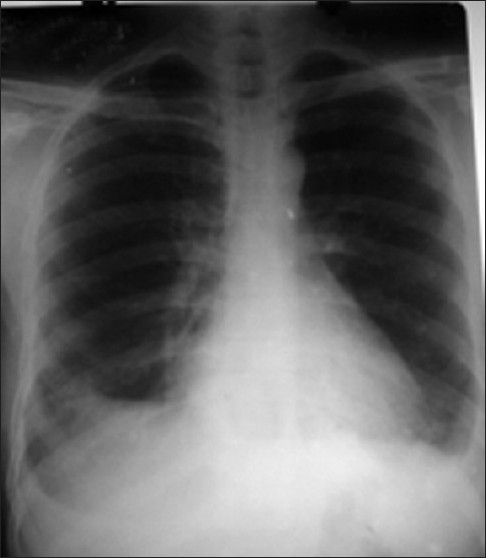
The chest X-ray after he presented to us showed homogenous opacities with bilateral pleural effusion

**Figure 2:**
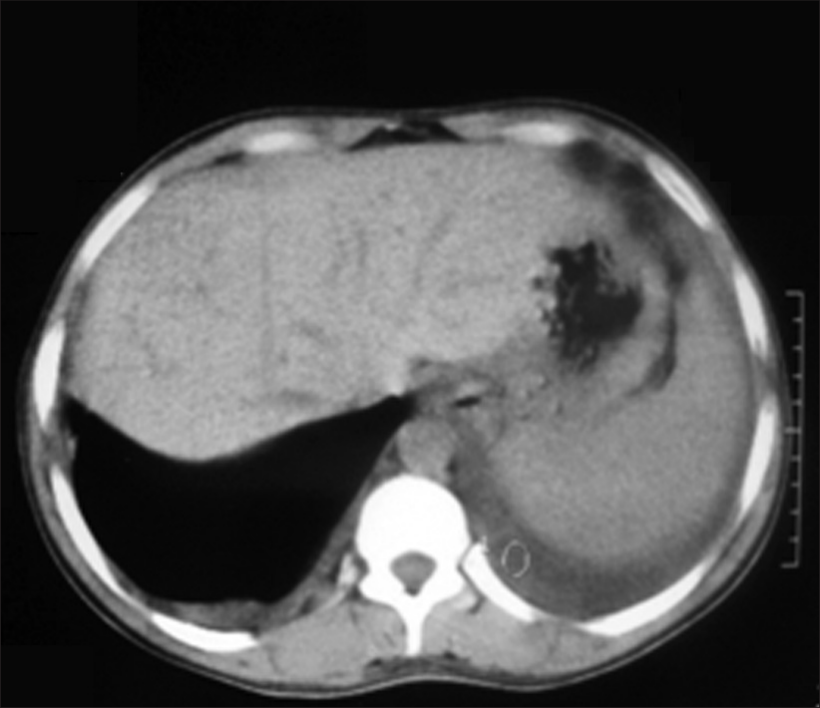
Bilateral pleural effusion and nodular pleural thickening in right lower lobe

The hemogram (hemoglobin level, RBC count, platelet count) except for TLC, coagulogram (APTT, INR, fibrin degradation product level), and routine blood biochemistry (serum urea, creatine, random blood sugar) were within normal limits. His total leucocyte count (TLC) at admission was 14 400/cu.mm. An arterial blood gas analysis revealed arterial oxygen tension (*p*O_2_) 78 mmHg, *p*CO _2_40 mmHg, HCO_3_25 mEq/l, and pH 7.38. Chest X-ray showed bilateral homogenous opacities with bilateral pleural effusion [Figure 1].The ultrasound thorax revealed 250 ml pleural fluid in the right pleural cavity and 150 ml in the left pleural cavity. A contrast enhanced CT thorax at admission showed bilateral pleural effusion with consolidation in the basal segment of both the lower lobes and nodular pleural thickening in the right lower lobe [Figure 2].

Diagnostic USG guided thoracocentesis of both the sides revealed hemorrhagic effusion on gross appearance. Biochemical analysis revealed them to as exudative in nature with ADA values of 9 IU/ml and 12 IU/ml of left and right side, respectively. Microscopic examination of pleural fluid revealed plenty of RBCs and 100% differential polymorph count in the left sided effusion and 80% differential polymorph count in the right-sided effusion. They were also negative for AFB or any microorganism on smears. Fibreoptic bronchoscopy revealed generalised hyperaemia of the tracheobronchial tree with pus oozing out from the more distal airways bilaterally. ZN staining of BAL fluid was negative for AFB. However, stained smear examination of BAL showed plenty of RBCs and neutrophils. Pleural fluid and BAL samples were sterile on culture.

**Figure 3:**
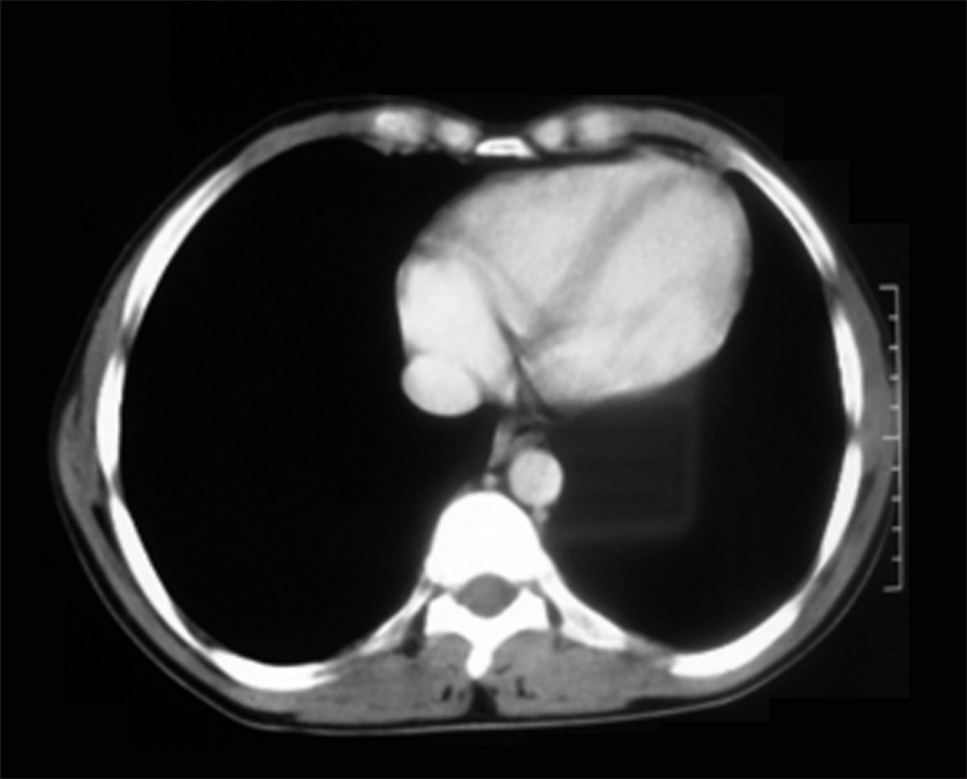
A repeat CT thorax shows the effusion has resolved following conservative management

The patient was treated with oxygen supplementation, oral corticosteroids (prednisolone at 1 mg/kg body weight) and injection ceftriaxone given 1 g thrice a day by intravenous route. The patient responded to the treatment and became afebrile with improvement in breathlessness and pleuritic chest pain. His TLC came down to 8000/cu.mm after 7 days of treatment. A repeat CT thorax done after 15 days showed resolution in the areas of consolidation and effusion [Figure 3]. Antibiotics and steroids after tapering were stopped after 3 weeks. The patient was discharged in a stable condition.

## DISCUSSION

Hydrocarbons are heterogeneous group of substances that are primarily composed of carbon and hydrogen molecules. They are quite abundant in modern society. Some of the most commonly used hydrocarbons include gasoline, lubricating oil, motor oil, mineral spirits, lighter fluid/naphtha, lamp oil, and kerosene.[1] Hydrocarbons can be classified as being aliphatic (ethane and acetylene), aromatic (toluene and phenol), or halogenated hydrocarbons which are a subgroup of aromatic hydrocarbons (chloroform and carbon tetra chloride).

Kerosene is a thin, clear liquid formed from a complex mixture of hydrocarbons, with density of 0.78–0.81 g/cm^3^. It is obtained from the fractional distillation of petroleum between 150 and 275°C, resulting in a mixture of carbon chains that typically contain between 12 and 15 carbon atoms per molecule.[2] In India, kerosene is the main fuel used for cooking and lighting among the poor. The lethal dose of kerosene for a 70 kg adult is 100 ml.[3]

Hydrocarbon exposure can be divided into the following four broad categories: nonintentional nonoccupational (or accidental, as occurred in our case), recreational, occupational, and intentional exposure. Kerosene poisoning is rare among adults.[4] It is a common practice in India for traders to buy kerosene in drums in bulk and then sell them in small amounts to individuals who bring their own jerry cans. To take out the kerosene from the drum, a vacuum is created in the hose by siphoning out the air using one’s mouth. Our patient mistook the amount of negative pressure required and ended up swallowing about 50 ml of kerosene after which he had an episode of vomiting and aspiration of the vomitus. Over the next 3 days he had nausea but no vomiting. Nausea subsided after that.

Signs of oral kerosene poisoning include diarrhea, nausea, and vomiting. The most frequent adverse effect of any hydrocarbon poisoning is aspiration, which can cause a chemical pneumonitis from direct injury to the lung parenchyma.[5] Pneumonia in the most cases of aspiration of hydrocarbons like Kerosene is interstitial and bilateral.[6] This damage depends on the viscosity (the resistance to flow, measured in Saybolt Seconds Universal [SSU]); volatility (the propensity to vaporize) and the chemical side chains of the hydrocarbon. Among them, viscosity is the single most important determinant of aspiration risk. Lower viscosity, especially less than 60 SSU and higher volatility are associated with a greater chance of aspiration with resultant pulmonary injury. The type II pneumocytes are the most affected resulting in decreased surfactant production. This decrease in surfactant results in alveolar collapse, ventilation – perfusion mismatch and hypoxemia. Hemorrhagic alveolitis can occur which peaks 3 days after ingestion. The end result of hydrocarbon aspiration is interstitial inflammation, intraalveolar hemorrhage and edema, hyperaemia, bronchial necrosis, and vascular necrosis.[7] The hemorrhagic alveolitis and bronchial and vascular necrosis can result in a hemorrhagic pleural effusion, which has been rarely reported.[8] Rare pulmonary complications include the development a pneumothorax, pneumatocele, or bronchopleural fistula. A rare complication of kerosene intoxication is cardiac arrhythmia and ventricular fibrillation, attributed to increased myocardial sensitivity to endogenous catecholamines.[9] Gastrointestinal involvement, as observed in our patient, is manifested by vomiting, abdominal pain and diarrhea has been attributed to mucosal irritation.[10] Symptoms and radiological findings resolve rapidly after cessation of exposure and corticosteroid therapy.[3]

To the best of our knowledge, our patient who developed bilateral hemorrhagic pleural effusion following aspiration of Kerosene while trying to siphon it out is the first reported case in Indian literature. The patient recovered satisfactorily following initial treatment consisting of oxygen inhalation, antibiotics, steroids, and supportive measures without any residual pulmonary sequel.
